# Explainable Machine Learning Models for Classifying Preoperative Anxiety in Patients With Femoral Neck Fractures: A Retrospective Cohort Study

**DOI:** 10.1002/nop2.70919

**Published:** 2026-10-16

**Authors:** Xue Zhang, Shuzhi Zheng, Zanhua Zhang, Peiyi Yang, Qiao Wei, Mimi Jin, Fang He

**Affiliations:** ^1^ Department of Post‐Anesthesia Care Unit The Second Affiliated Hospital of Wenzhou Medical University Wenzhou Zhejiang China; ^2^ Department of Operating Room The Second Affiliated Hospital of Wenzhou Medical University Wenzhou Zhejiang China; ^3^ Department of Intensive Care Unit The Second Affiliated Hospital of Wenzhou Medical University Wenzhou Zhejiang China; ^4^ Department of General Intensive Care Unit The Second Affiliated Hospital of Wenzhou Medical University Wenzhou Zhejiang China

**Keywords:** explainable artificial intelligence, femoral neck fracture, internal validation, machine learning, perioperative nursing, preoperative anxiety

## Abstract

**Aim:**

To develop and internally validate explainable machine learning models for concurrent classification of preoperative anxiety in patients with femoral neck fractures.

**Design:**

Single‐centre retrospective observational cohort study.

**Methods:**

Clinical, perioperative and psychosocial data from 271 surgical admissions (270 patients) during 2024 were analysed. Preoperative anxiety was classified during the preoperative bedside assessment phase using a prespecified score greater than 8 on the anxiety subscale of the Hospital Anxiety and Depression Scale. Three tree‐based ensemble models were compared using fivefold, patient‐grouped nested cross‐validation with fold‐specific preprocessing, feature selection, resampling, tuning and separate probability calibration.

**Results:**

Preoperative anxiety according to this prespecified definition was identified in 79 admissions. The anxiety group was older, had lower psychological resilience and perceived social support, and had greater surgical fear and frailty. The three algorithms showed similar internal discrimination, and paired comparisons did not support superiority of one model. Psychological resilience, perceived social support and surgical fear were selected in every validation fold for all models. Frailty index was selected in three of five folds for each model.

**Conclusion:**

The models identified a stable psychosocial feature profile for concurrent classification but require external and prospective validation before clinical use.

**Implications for Nursing Practice:**

These findings may inform future research on structured nursing assessment of preoperative anxiety. The models should not replace validated screening or clinical judgement and require prospective evaluation before routine use.

**Impact:**

Psychological resilience, perceived social support and surgical fear were the most stable selected features.

**Reporting Method:**

The Strengthening the Reporting of Observational Studies in Epidemiology and TRIPOD+AI reporting guidelines were followed.

**No Patient or Public Contribution:**

None, because this study retrospectively analysed existing de‐identified records.

## 1 Introduction

Recovery after femoral neck fracture may be complex and non‐linear (Olofsson et al. 2026). The acute fracture and planned surgery can be accompanied by pain, uncertainty and concern about recovery. For perioperative nurses, preoperative anxiety is clinically relevant because it can affect communication, sleep, cooperation with preparation and postoperative recovery behaviours. In older fracture patients, anxiety assessment is also complicated by frailty, comorbidity, reduced mobility and variable health literacy.

Current bedside assessment of preoperative anxiety is usually based on patient report, clinical observation or screening scales. These approaches remain necessary, but they may not integrate physical, psychological and social factors into a reproducible risk estimate. Anxiety and depressive symptoms have been documented before and after total hip or knee arthroplasty (de Filippis et al. 2024). These symptoms have also been associated with infection, revision and reoperation (Harmer et al. 2023). A systematic review identified associations between anxiety, depression or pain catastrophizing and postoperative arthroplasty outcomes (Wood et al. 2021). However, evidence specific to femoral neck fracture patients is limited, and the available literature does not establish whether a prediction model improves care.

Machine learning can model non‐linear relations between patient characteristics and binary outcomes, but predictive accuracy alone is insufficient for nursing use. Reviews of nursing artificial intelligence (AI) have described contributions to decision‐making (Fernandes et al. 2026) and a heterogeneous evidence base for AI‐based technologies in nursing (von Gerich et al. 2022). Models intended for clinical interpretation should also report the data source, outcome definition, validation approach and feature contribution method. Explainable AI methods, including Shapley additive explanations (SHAP), can describe the direction and relative contribution of predictors, but they do not by themselves test effects on patient assessment or outcomes. This study therefore focused on model development, internal validation and interpretation rather than implementation testing.

## 2 Background

The biopsychosocial model provides a basis for combining physical vulnerability, psychological state and social context in perioperative risk assessment (Bolton 2023). Frailty is relevant to perioperative assessment in older surgical patients (Boccardi and Marano 2024). Psychological resilience has been described as an emergent characteristic (Tay and Lim 2020) and has been associated with physical function after hip fracture surgery (Lim et al. 2020). Postoperative kinesiophobia in older patients with femoral neck fracture has also been linked to psychological and social determinants (Guo et al. 2025). In this study, physiological vulnerability was represented by frailty and comorbidity measures, psychological factors by resilience and surgical fear, and social context by perceived social support and familiar faces at admission. This framework was used to organise variables rather than to infer causality.

The research gap addressed by this study is the lack of an internally validated and interpretable model for concurrent classification of preoperative anxiety in this fracture population. The study aimed to determine whether a combined biopsychosocial feature set could classify preoperative anxiety defined using the Hospital Anxiety and Depression Scale anxiety subscale, compare three tree‐based ensemble methods under the same validation framework, and describe the stability and direction of predictor contributions.

## 3 The Study

### 3.1 Aim

The aims were to develop and internally validate machine learning models for concurrent classification of preoperative anxiety in patients with femoral neck fractures, compare model performance under a common patient‐grouped nested cross‐validation framework, and describe stable predictors and Extreme Gradient Boosting (XGBoost) feature contributions.

## 4 Methods

### 4.1 Design

This was a single‐centre retrospective observational cohort study conducted at the Second Affiliated Hospital of Wenzhou Medical University, Wenzhou, China, between 1 January and 31 December 2024. The unit of analysis was the surgical admission. The dataset contained 271 eligible admissions involving 270 patients, and patient‐grouped cross‐validation assigned records from the same patient to the same outer fold. The records had been obtained before the present analysis and were reviewed after de‐identification. The study followed the Strengthening the Reporting of Observational Studies in Epidemiology and TRIPOD+AI reporting guidelines (von Elm et al. 2007).

### 4.2 Sampling and Recruitment

Eligible participants were adults aged 18 years or older with imaging‐confirmed acute femoral neck fracture who were scheduled for surgery and were able to complete bedside communication and questionnaire assessment. Patients were excluded for pathological fracture or high‐energy multiple trauma, severe cognitive impairment, severe psychiatric disorder or long‐term psychotropic medication use, acute preoperative delirium, conservative treatment or surgical contraindication, or more than 20% missing information among core indicators.

### 4.3 Instruments With Validity and Reliability

The primary outcome was preoperative anxiety assessed using the anxiety subscale of the Hospital Anxiety and Depression Scale (HADS‐A) (Zigmond and Snaith 1983). In the original data‐collection protocol, significant preoperative anxiety was defined a priori as a HADS‐A score > 8, consistent with an approach used in prior arthroplasty research (de Filippis et al. 2024). Only the resulting binary classification (0 = no, 1 = yes) was entered into the final analytic file; the underlying HADS‐A total scores and item‐level responses were not recorded in that file and therefore could not be reconstructed, reclassified using alternative cutoffs or used for a score‐based sensitivity analysis. Assessments were performed 24–48 h after admission and at least 12 h before surgery by trained researchers using face‐to‐face bedside interviews.

### 4.4 Sample Size and Power

All eligible admissions during the prespecified 2024 period were included. No a priori sample‐size or power calculation was performed because the study used an existing retrospective cohort. The analytic cohort contained 271 admissions and 79 anxiety events. Given the limited event count, the analyses were treated as exploratory; optimism was addressed using patient‐grouped nested cross‐validation and uncertainty intervals were estimated using patient‐cluster bootstrap resampling.

### 4.5 Data Sources and Collection

Candidate variables were obtained from the electronic health record and bedside questionnaires and organised according to a biopsychosocial framework. Clinical variables included age, gender, body mass index, haemoglobin, albumin, Charlson comorbidity index, American Society of Anesthesiologists (ASA) grade, comorbidities, injury‐to‐admission time, surgical and anaesthetic history and preoperative pain score. Psychosocial variables included the Connor–Davidson Resilience Scale (CD‐RISC) (Connor and Davidson 2003), Surgical Fear Questionnaire (SFQ) (Theunissen et al. 2014), Multidimensional Scale of Perceived Social Support (MSPSS) (Zimet et al. 1988), familiar faces at admission and health literacy–related measures. Frailty was assessed using a Frailty index and a categorical frailty status variable. The cited original publications describe the development or validation of HADS‐A, CD‐RISC, SFQ and MSPSS (Zigmond and Snaith 1983; Connor and Davidson 2003; Theunissen et al. 2014; Zimet et al. 1988). For CD‐RISC, SFQ and MSPSS, the submitted analytic file contained scale totals rather than item‐level responses; HADS‐A was available only as the binary classification described above. Study‐specific internal‐consistency coefficients therefore could not be calculated.

### 4.6 Data Analysis

The final analytic dataset contained 271 admissions and 40 recorded variables. The intended model time point was the preoperative bedside assessment phase. Candidate clinical and questionnaire variables were obtained from records compiled around that phase; however, the source records did not preserve a field‐level timestamp for every candidate variable. Case Order and OR Entry Time were excluded because they were administrative or operational fields with unclear availability at the intended assessment time. Serial number, identification number, patient identifier, name and surgery date were also excluded, as was the outcome preoperative anxiety, leaving 32 numeric candidate predictors. HADS‐A and several psychosocial measures were collected during the same preoperative assessment phase. The analysis therefore evaluates concurrent classification, not prediction of a later independent outcome. No outcome or candidate‐predictor values were missing or infinite. A median imputer and standardisation step were retained in the pipeline and fitted separately within each inner training fold; because the analytic dataset had no missing predictor values, the imputer did not replace any observations. Least absolute shrinkage and selection operator (LASSO) logistic regression with L1 regularisation was used for feature reduction (Tibshirani 1996). Its inverse‐regularisation parameter was tuned within the inner grouped cross‐validation, and variables with absolute non‐zero coefficients greater than 1 × 10^−6^ were retained. This procedure was repeated independently in each outer fold; no single fixed feature set was imposed on all validation folds. The data and predictor‐timing audits are reported in File S1 (Tables S1 and S8).

### 4.7 Model Development and Validation

Three tree‐based ensemble models were compared: XGBoost (Chen and Guestrin 2016), random forest (Breiman 2001) and Light Gradient Boosting Machine (LightGBM) (Ke et al. 2017). Random forest represented bagging, whereas XGBoost and LightGBM represented gradient boosting. These models were selected to compare bagging and boosting approaches that can accommodate non‐linear relations between the candidate variables and concurrent classification outcome; their tree structures also permit Shapley‐based feature‐contribution analysis. XGBoost was used for out‐of‐fold TreeSHAP analysis as a representative boosted‐tree model and was not assumed superior. Fivefold stratified group cross‐validation was used for outer validation, with patient identifier as the grouping variable and random seed 2022. In each outer training set, approximately 75% of records were used for model fitting and 25% for Platt sigmoid calibration (Niculescu‐Mizil and Caruana 2005). Fourfold stratified group cross‐validation and 18 random hyperparameter combinations were used within the fitting subset. Each inner training pipeline refitted median imputation, standardisation, LASSO selection and synthetic minority oversampling technique (SMOTE) resampling (Chawla et al. 2002) before estimating model parameters. No class‐weight parameter was applied after SMOTE. Search ranges were comparable in scale: XGBoost and LightGBM used 100–350 trees and learning rates of 0.02–0.10; random forest used 300–800 trees, maximum depths of 3, 4 or 6, and minimum leaf sizes of 2, 4 or 8. Other depth, sampling, regularisation and leaf‐size parameters were tuned within prespecified ranges. The outer test fold was not used for preprocessing, feature selection, resampling, tuning or calibration. Search ranges and outer‐fold partitions are reported in File S1 (Tables S2 and S3).

### 4.8 Performance, Calibration and Interpretability

Out‐of‐fold probabilities were assembled so that each admission was predicted only by a model that had not used that patient for model fitting, tuning or calibration. Discrimination was assessed using the area under the receiver operating characteristic curve (AUC). Calibration was assessed using Brier score, calibration intercept, calibration slope and an eight‐bin quantile calibration plot. Uncertainty was estimated with 2000 patient‐cluster bootstrap resamples of the complete out‐of‐fold prediction set; paired bootstrap differences were used for model comparisons. Decision curve analysis evaluated net benefit over threshold probabilities (Vickers and Elkin 2006). Patient‐cluster bootstrap pointwise 95% confidence intervals were calculated for the three model curves and the treat‐all curve; the treat‐none line was the theoretical zero‐net‐benefit reference. Probability tertiles were calculated descriptively from random‐forest out‐of‐fold predictions and were not treated as clinical thresholds. XGBoost feature contributions were calculated for each outer test fold using TreeSHAP and then combined across folds (Lundberg and Lee 2017). The previous auxiliary four‐variable scoring‐weight figure was removed because it was not independently validated and did not represent the fitted tree models.

### 4.9 Ethical Considerations

The retrospective analysis used clinical and preoperative questionnaire records collected in 2024; the data were de‐identified before analysis. An Ethics Committee archival‐check note concerning the 2024 preoperative HADS‐A data records that the 2024 protocol covered these assessments, that the recorded consent arrangement was written informed consent, and that use of the data for this retrospective analysis and publication was within the existing authorization. A separate approval was granted after data collection by the Ethics Committee of the Second Affiliated Hospital of Wenzhou Medical University and Yuying Children's Hospital of Wenzhou Medical University (No. 2026‐K‐343‐01; review date: 21 June 2026). The official stamped archival‐check document identifies this decision as an initial review. This decision was issued after data collection and is not presented as the basis for authorization of the 2024 data collection or use. Access to the analysis dataset was restricted to authorised study‐team members.

## 5 Results

### 5.1 Participants

A total of 418 surgical admission records for adults with femoral neck fractures were screened. After exclusion of 147 records, 271 surgical admissions involving 270 patients were included in the final analysis. Exclusions were due to pathological fracture or high‐energy multiple trauma (*n* = 24), severe psychiatric disorder or long‐term psychotropic medication use (*n* = 15), severe cognitive impairment (*n* = 65), and incomplete core clinical or questionnaire data (*n* = 43). According to the prespecified HADS‐A > 8 definition, 79 admissions (29.2%) were classified as having preoperative anxiety and 192 admissions (70.8%) as not having preoperative anxiety. The participant flow is shown in Figure 1.

**FIGURE 1:**
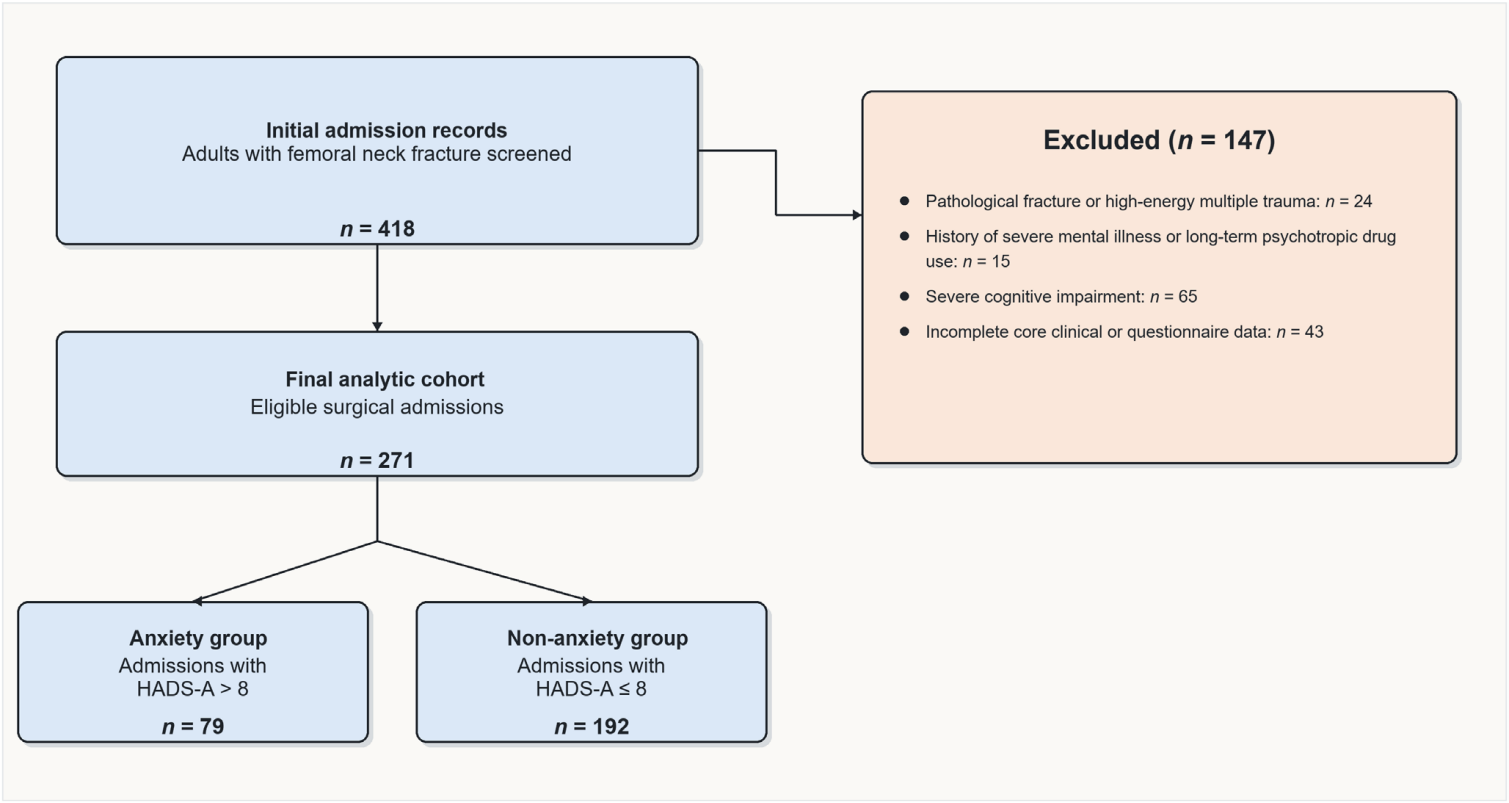
Participant flow diagram. The flow diagram shows record screening, exclusion and final cohort inclusion. A total of 418 surgical admission records for adults with femoral neck fractures were screened; 147 were excluded because of pathological fracture or high‐energy multiple trauma (*n* = 24), severe psychiatric disorder or long‐term psychotropic medication use (*n* = 15), severe cognitive impairment (*n* = 65), or incomplete core clinical or questionnaire data (*n* = 43). The final analytic cohort included 271 surgical admissions; 79 admissions met the prespecified HADS‐A > 8 definition of preoperative anxiety and 192 did not. HADS‐A, anxiety subscale of the Hospital Anxiety and Depression Scale.

Baseline comparisons are shown in Tables 1, 2, 3. The anxiety group was older than the non‐anxiety group (70.39 ± 8.63 vs. 65.48 ± 8.90 years, *p* < 0.001). Albumin was lower in the anxiety group (39.25 ± 4.22 vs. 40.66 ± 4.06 g/L, *p* = 0.011). The preoperative Visual Analogue Scale (VAS) score did not differ significantly between groups (3.81 ± 1.54 vs. 3.59 ± 1.78, *p* = 0.346).

**TABLE 1:** Baseline demographic characteristics.

Variable	Non‐anxiety grou*p* (*n* = 192)	Anxiety grou*p* (*n* = 79)	*p*
Age (years)	65.48 ± 8.90	70.39 ± 8.63	< 0.001
Height (cm)	161.64 ± 7.16	161.22 ± 7.48	0.669
Weight (kg)	59.00 ± 9.85	58.65 ± 11.01	0.794
BMI (kg/m^2^)	22.64 ± 3.88	22.60 ± 4.19	0.936
Injury‐to‐admission interval (days)	1.22 ± 1.27	1.32 ± 1.50	0.572
Gender, *n* (%)			0.928
Female	126 (65.6%)	53 (67.1%)	
Male	66 (34.4%)	26 (32.9%)	
Education level, *n* (%)			0.413
Primary school or below	145 (75.5%)	64 (81.0%)	
Above primary school	47 (24.5%)	15 (19.0%)	
Payment method, *n* (%)			0.409
Social security	67 (34.9%)	25 (31.6%)	
Agricultural insurance	61 (31.8%)	21 (26.6%)	
Self‐pay	64 (33.3%)	33 (41.8%)	

*Note:* Values are mean ± standard deviation (SD) or *n* (%). *p* values compare admissions with and without prespecified HADS‐A‐defined preoperative anxiety.

Abbreviations: BMI, body mass index; HADS‐A, anxiety subscale of the Hospital Anxiety and Depression Scale; kg/m^2^, kilograms per square metre.

**TABLE 2:** Clinical indicators and comorbidities.

Variable	Non‐anxiety grou*p* (*n* = 192)	Anxiety grou*p* (*n* = 79)	*p*
Haemoglobin (Hb, g/L)	126.86 ± 19.65	127.87 ± 18.36	0.696
Albumin (Alb, g/L)	40.66 ± 4.06	39.25 ± 4.22	0.011
ASA grade, *n* (%)			0.077
Grade I	3 (1.6%)	2 (2.5%)	
Grade II	173 (90.1%)	63 (79.7%)	
Grade III	16 (8.3%)	13 (16.5%)	
Grade IV	0 (0.0%)	1 (1.3%)	
Hypertension, *n* (%)	107 (55.7%)	49 (62.0%)	0.413
Diabetes, *n* (%)	30 (15.6%)	14 (17.7%)	0.807
Heart disease, *n* (%)	19 (9.9%)	9 (11.4%)	0.882
Brain disease, *n* (%)	23 (12.0%)	9 (11.4%)	1.000
Kidney disease, *n* (%)	11 (5.7%)	9 (11.4%)	0.172
Osteoporosis, *n* (%)	81 (42.2%)	41 (51.9%)	0.185
Previous surgery/anaesthesia, *n* (%)	61 (31.8%)	31 (39.2%)	0.589

*Note:* Values are mean ± standard deviatio*n* (SD) or *n* (%).

Abbreviations: Alb, albumin; ASA, American Society of Anesthesiologists; CCI, Charlson comorbidity index; Hb, haemoglobin.

**TABLE 3:** Psychological and functional assessment scores.

Variable	Non‐anxiety grou*p* (*n* = 192)	Anxiety grou*p* (*n* = 79)	*p*
Preoperative VAS score	3.59 ± 1.78	3.81 ± 1.54	0.346
Visual health disturbance score	52.91 ± 15.51	57.00 ± 19.65	0.070
CD‐RISC score	64.36 ± 12.53	48.66 ± 13.34	< 0.001
SFQ score	8.08 ± 3.71	11.47 ± 3.82	< 0.001
MSPSS score	57.80 ± 12.75	48.05 ± 9.67	< 0.001
Frailty index	0.20 ± 0.08	0.26 ± 0.08	< 0.001
Familiar faces present, *n* (%)	103 (53.6%)	26 (32.9%)	0.003
Frailty status, *n* (%)			< 0.001
Non‐frail	22 (11.5%)	4 (5.1%)	
Pre‐frail	123 (64.1%)	36 (45.6%)	
Frail	47 (24.5%)	39 (49.4%)	

*Note:* Values are mean ± standard deviatio*n* (SD) or *n* (%). Visual health disturbance score is the health‐literacy‐related measure recorded in the submitted dataset.

Abbreviations: CD‐RISC, Connor‐Davidson Resilience Scale; MSPSS, Multidimensional Scale of Perceived Social Support; SFQ, Surgical Fear Questionnaire; VAS, visual analogue scale.

Psychosocial and frailty‐related variables differed between groups. Compared with the non‐anxiety group, the anxiety group had lower CD‐RISC scores (48.66 ± 13.34 vs. 64.36 ± 12.53, *p* < 0.001), lower MSPSS scores (48.05 ± 9.67 vs. 57.80 ± 12.75, *p* < 0.001), higher SFQ scores (11.47 ± 3.82 vs. 8.08 ± 3.71, *p* < 0.001), and a higher Frailty index (0.26 ± 0.08 vs. 0.20 ± 0.08, *p* < 0.001).

### 5.2 Feature Selection

Feature selection was repeated within each outer training fold. CD‐RISC, MSPSS and SFQ were retained in all five outer folds for XGBoost, random forest and LightGBM. Frailty index was retained in three of five folds for each model. Preoperative VAS was retained in three of five folds for XGBoost and LightGBM and in two of five random‐forest folds despite a non‐significant unadjusted group comparison. This difference reflects the distinct purposes of marginal comparison and penalised multivariable classification; selection does not establish an independent association or conventional statistical significance. Feature‐selection frequencies are reported in File S1 (Table S6), and the selection patterns are shown in Figure 2.

**FIGURE 2:**
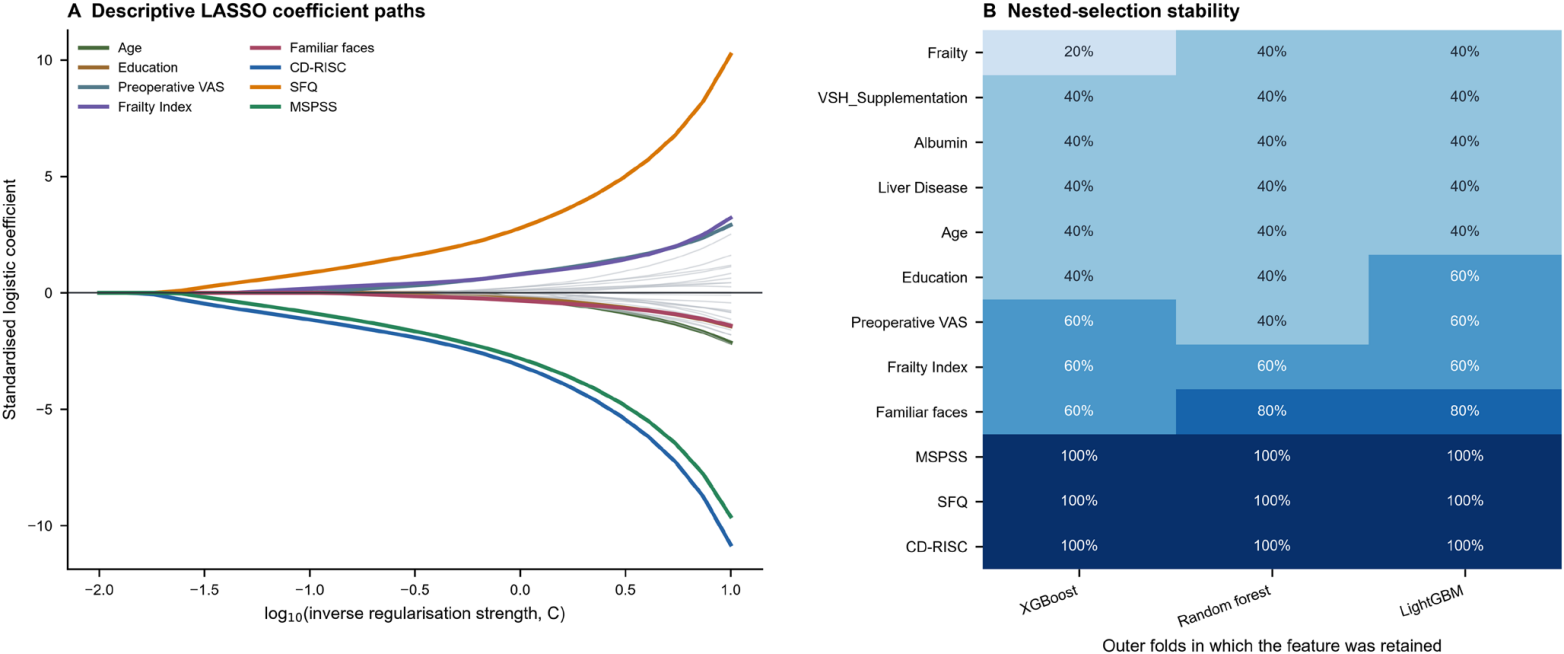
Least absolute shrinkage and selection operator (LASSO) coefficient paths and nested‐selection stability. (A) Descriptive standardised LASSO coefficient paths in the full analytic dataset; these paths are presented for visual orientation and were not used to estimate validation performance. (B) The proportion of the five outer folds in which each of the 12 most frequently retained predictors was selected for XGBoost, random forest and LightGBM. All preprocessing and LASSO selection used for validation were repeated within inner training folds. ASA, American Society of Anesthesiologists; C, inverse regularisation strength; CD‐RISC, Connor‐Davidson Resilience Scale; HLS, health‐literacy score; LASSO, least absolute shrinkage and selection operator; MSPSS, Multidimensional Scale of Perceived Social Support; SFQ, Surgical Fear Questionnaire; VAS, visual analogue scale.

### 5.3 Model Performance

The three models showed similar out‐of‐fold discrimination (Table 4 and Figure 3). AUC was 0.921 (95% confidence interval [CI] 0.885–0.953) for XGBoost, 0.932 (95% CI 0.900–0.959) for random forest and 0.928 (95% CI 0.894–0.957) for LightGBM. The paired AUC difference for random forest minus XGBoost was 0.011 (95% CI −0.011 to 0.033), for LightGBM minus XGBoost was 0.007 (95% CI −0.013 to 0.029), and for LightGBM minus random forest was −0.004 (95% CI −0.027 to 0.019). Corresponding Brier scores were 0.099 (95% CI 0.074–0.124), 0.096 (95% CI 0.075–0.118) and 0.096 (95% CI 0.074–0.119). Random‐forest out‐of‐fold predictions had a calibration intercept of 0.041 (95% CI −0.325 to 0.386) and calibration slope of 1.017 (95% CI 0.690–1.571). The overlapping intervals and paired comparisons do not support superiority of one algorithm in this dataset. Detailed out‐of‐fold estimates and paired model comparisons are reported in File S1 (Tables S4 and S5).

**TABLE 4:** Patient‐grouped nested cross‐validation performance for concurrent preoperative anxiety classification.

Model	AUC (95% CI)	Brier score (95% CI)	Calibration intercept (95% CI)	Calibration slope (95% CI)
XGBoost	0.921 (0.885–0.953)	0.099 (0.074–0.124)	0.028 (−0.397 to 0.447)	0.907 (0.746–1.168)
Random forest	0.932 (0.900–0.959)	0.096 (0.075–0.118)	0.041 (−0.325 to 0.386)	1.017 (0.690–1.571)
LightGBM	0.928 (0.894–0.957)	0.096 (0.074–0.119)	−0.053 (−0.452 to 0.309)	1.056 (0.847–1.355)

*Note:* Metrics were calculated from out‐of‐fold probabilities generated by fivefold stratified, patient‐grouped nested cross‐validation for concurrent classification during the preoperative assessment phase. Confidence intervals are 2000‐resample patient‐cluster percentile bootstrap intervals. Pairwise AUC‐difference confidence intervals all included zero.

Abbreviations: AUC, area under the receiver operating characteristic curve; CI, confidence interval.

**FIGURE 3:**
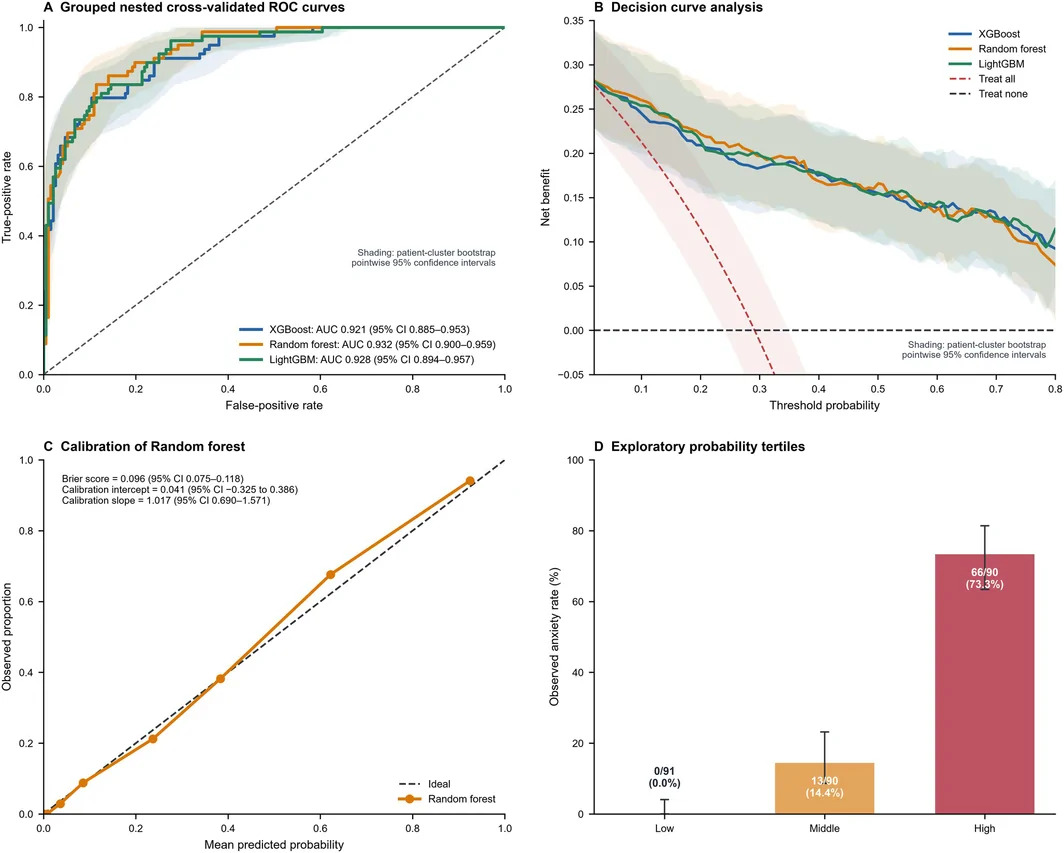
Patient‐grouped nested cross‐validation performance. (A) Receiver operating characteristic curves from the combined out‐of‐fold predictions. The legend reports patient‐cluster bootstrap 95% confidence intervals for area under the curve. The shaded regions are pointwise 95% confidence intervals for sensitivity at a common false‐positive rate grid, estimated from 2000 patient‐cluster bootstrap resamples; they are not simultaneous confidence bands. (B) Decision curves calculated from the same out‐of‐fold probabilities, with patient‐cluster bootstrap pointwise 95% confidence intervals for the three model curves and the treat‐all curve; the treat‐none line is the theoretical zero‐net‐benefit reference. (C) The eight‐bin quantile calibration plot for random forest, which had the highest AUC point estimate; its Brier score, calibration intercept and calibration slope are printed with patient‐cluster bootstrap 95% confidence intervals in the panel. (D) Observed anxiety rates with Wilson 95% confidence intervals across descriptive tertiles of random‐forest out‐of‐fold probabilities. These tertiles are not clinical thresholds. AUC, area under the receiver operating characteristic curve.

### 5.4 Exploratory Probability‐Tertile Analysis

Using random‐forest out‐of‐fold probabilities, observed anxiety occurred in 0/91 admissions (0.0%; 95% CI 0.0%–4.1%) in the low tertile, 13/90 (14.4%; 95% CI 8.6%–23.2%) in the middle tertile, and 66/90 (73.3%; 95% CI 63.4%–81.4%) in the high tertile. Corresponding probability ranges were 0.0000–0.0435, 0.0438–0.3687 and 0.3688–1.0000. These sample‐dependent tertiles describe internal classification ordering and are not validated clinical cut‐off points. Detailed tertile results are reported in File S1 (Table S7).

### 5.5 Model Interpretability

Out‐of‐fold XGBoost TreeSHAP analysis identified CD‐RISC, MSPSS, SFQ and Frailty index as the four leading contributors by mean absolute contribution. Higher CD‐RISC and MSPSS values generally produced negative contributions, indicating a lower XGBoost‐predicted probability of anxiety. Higher SFQ values and, less consistently, higher Frailty index values produced positive contributions. CD‐RISC, MSPSS and SFQ were also retained in every outer fold for all three models. The out‐of‐fold TreeSHAP summaries are shown in Figure 4.

**FIGURE 4:**
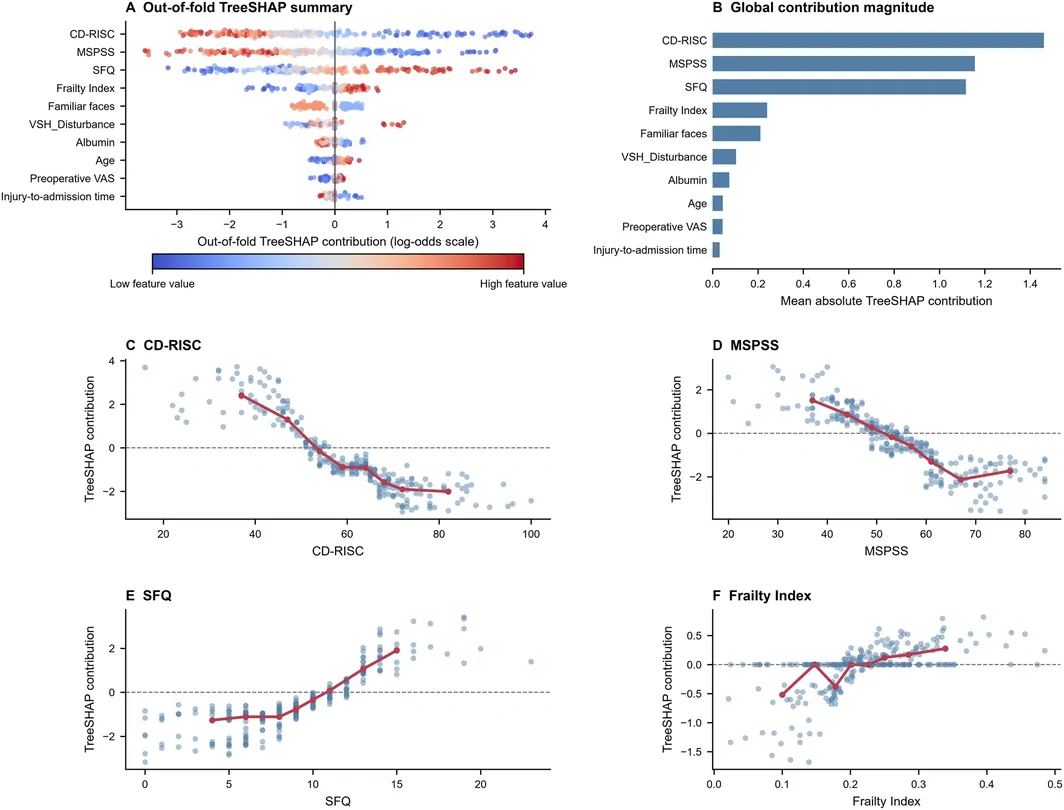
Out‐of‐fold XGBoost tree‐based Shapley additive explanations (TreeSHAP). (A) Combines tree‐based Shapley additive explanation contributions calculated in each outer test fold. (B) Ranks features by mean absolute out‐of‐fold contribution. (C–F) Relate feature values to contributions for CD‐RISC, MSPSS, SFQ and Frailty index; red lines show descriptive bin‐specific medians. Higher CD‐RISC and MSPSS values generally contributed to lower XGBoost‐estimated probabilities of anxiety, whereas higher SFQ values and, less consistently, higher Frailty index values contributed to higher estimated probabilities of anxiety. CD‐RISC, Connor‐Davidson Resilience Scale; MSPSS, Multidimensional Scale of Perceived Social Support; SFQ, Surgical Fear Questionnaire; TreeSHAP, tree‐based Shapley additive explanations.

TreeSHAP values describe model behaviour rather than causal effects or a clinical score. The auxiliary scoring‐weight figure used in a previous draft was removed because it was based on a separate logistic regression and had not undergone independent validation.

## 6 Implications for Nursing Practice

The present results do not support implementation of a model in routine nursing practice. Future prospective nursing studies should define a pre‐HADS‐A assessment time point, collect candidate predictors before outcome screening, prespecify models and action thresholds, and evaluate feasibility, subgroup calibration, algorithmic bias, clinician oversight and patient‐centred outcomes. Until such studies are completed, structured assessment should continue to use validated anxiety screening and clinical judgement.

## 7 Discussion

### 7.1 Principal Findings

This retrospective cohort study internally evaluated three machine learning models for concurrent classification of preoperative anxiety in patients with femoral neck fractures using patient‐grouped nested cross‐validation. Random forest had the highest AUC point estimate, but paired bootstrap intervals did not support superiority over XGBoost or LightGBM. The three models had AUCs of 0.921–0.932 and Brier scores of 0.096–0.099. A systematic review of orthopaedic surgery found substantial heterogeneity in the instruments and timing used to assess preoperative anxiety, with HADS being the most frequently used instrument (Opplert et al. 2025). Predictive modelling with XGBoost has also been reported for postoperative complications in orthopaedic patients (Huang et al. 2025). The stable feature profile included psychological resilience, perceived social support and surgical fear; XGBoost interpretation also identified frailty. These findings support a biopsychosocial approach to assessment but do not show effects on nursing workflow or patient outcomes.

### 7.2 Interpretation of Predictor Profile

The direction of the main predictors was clinically coherent. Lower CD‐RISC and MSPSS scores were observed in admissions with preoperative anxiety, while higher SFQ scores and higher Frailty index values were observed in the anxiety group. CD‐RISC, MSPSS and SFQ were selected in every outer fold for all three models. Preoperative VAS was retained in three of five XGBoost and LightGBM folds and in two of five random‐forest folds despite no significant unadjusted group difference. This indicates that VAS sometimes added information conditional on other variables; it does not establish an independent association, interaction, causal effect or stable clinical predictor. Prior hip‐fracture research has linked resilience with physical function (Lim et al. 2020). A prospective study in older patients with femoral neck fracture has described psychological and social determinants of postoperative kinesiophobia (Guo et al. 2025). Because the study was observational, predictor contributions should not be interpreted as causal effects.

### 7.3 Model Interpretation

Out‐of‐fold TreeSHAP analysis described how predictors contributed to XGBoost output without using the same observations for model fitting and interpretation. SHAP values describe model behaviour rather than causal effects or clinical validity, and explainability should not substitute for rigorous internal and external validation (Ghassemi et al. 2021). Calibration is also essential when predicted probabilities are interpreted (Van Calster et al. 2019). For nurses, these findings may support future research on structured discussion of risk factors during preoperative assessment, but they do not replace bedside judgement. A systematic review of clinician trust in AI‐based clinical decision support supports retaining human oversight (Tun et al. 2025). The unvalidated scoring‐weight display was removed rather than presented as a clinical aid.

### 7.4 Strengths and Limitations

The study combined clinical, frailty‐related and psychosocial variables and repeated preprocessing, feature selection, SMOTE, tuning and calibration within patient‐grouped nested validation. Several limitations remain. First, this was a single‐centre retrospective analysis with 79 events (Riley et al. 2019), and the limited event count, class imbalance and fold‐specific calibration can leave residual overfitting despite nested validation. The source records did not preserve field‐level acquisition times for every candidate predictor, and HADS‐A and several psychosocial measures were collected during the same preoperative assessment phase. The models therefore represent concurrent classification and cannot establish availability of predictors at a future decision point. Only the binary HADS‐A classification was retained in the analytic file; HADS‐A totals and items could not be verified against source records, reclassified using alternative thresholds or assessed in a sensitivity analysis. The model has not been externally or prospectively validated, and dataset shift, subgroup calibration and algorithmic bias were not evaluated. The findings do not establish clinical utility, nursing workflow benefit or patient benefit.

### 7.5 Recommendations for Further Research

Future studies should use prospectively collected data with a prespecified predictor‐availability schedule, retain HADS‐A totals and item responses where appropriate, externally validate calibration and discrimination, assess subgroup performance and algorithmic bias and test whether structured nursing assessment informed by the model changes patient‐centred outcomes.

## 8 Conclusion

In this retrospective cohort, three tree‐based models provided similar internal performance for concurrent classification of preoperative anxiety during the preoperative assessment phase. CD‐RISC, MSPSS and SFQ were stable selected features, but the models require prospective and external evaluation before they can inform clinical practice.

## Author Contributions

**Xue Zhang:** conceptualization, methodology, data curation, formal analysis, writing – original draft. **Shuzhi Zheng:** validation, formal analysis. **Zanhua Zhang:** data curation, investigation. **Peiyi Yang:** data curation, investigation. **Qiao Wei:** methodology, software. **Mimi Jin:** visualization, writing – review and editing. **Fang He:** validation, supervision, project administration, writing – review and editing.

## Funding

This work was supported by the Wenzhou Municipal Science and Technology Bureau (grant number RC20250015).

## Disclosure

Statistical Guidelines: Shuzhi Zheng, the second author, is the statistician on the author team and checked the analytic dataset and reported statistical results. The review covered variable definitions, predictor timing and availability, exclusion of administrative and operational fields, patient grouping, model outputs, confidence intervals, tables and figures. The analyses were repeated from the read‐only source file after Case Order and OR Entry Time were excluded. The authors affirm that the methods used in the data analyses are suitably applied to the data within the study design and context, and that the statistical findings have been implemented and interpreted correctly. Random seed 2022 was used.

Data Confidentiality and Access: Patient identifiers were removed before analysis. The analysis dataset was stored in a password‐protected local research workspace. Access was limited to authorised members of the study team who were responsible for data cleaning, statistical analysis and manuscript preparation. Patient‐level data are not publicly available because they contain clinical information from hospital records.

## Ethics Statement

This manuscript reports a retrospective analysis of clinical and preoperative questionnaire records collected in 2024, which were de‐identified before analysis. The Ethics Committee's archival‐check note records that the 2024 protocol covered the preoperative HADS‐A assessments, that the recorded consent arrangement was written informed consent, and that use of the data for this retrospective analysis and publication was within the existing authorization. A separate Ethics Committee approval (No. 2026‐K‐343‐01; review date: 21 June 2026) was granted after data collection. The official stamped archival‐check document identifies Ethics Committee decision No. 2026‐K‐343‐01 as an initial review (review date: 21 June 2026). This decision was issued after data collection and is not presented as the basis for authorization of the 2024 data collection or use.

## Consent

Patients or members of the public were not involved because this was a retrospective analysis of existing de‐identified records.

## Conflicts of Interest

The authors declare no conflicts of interest.

## Supporting information


**File S1:** Statistical Appendix.
**Table S1:** Data audit and analysis set.
**Table S2:** Prespecified model search ranges.
**Table S3:** Outer‐fold data partitions.
**Table S4:** Out‐of‐fold performance with patient‐cluster bootstrap confidence intervals.
**Table S5:** Paired out‐of‐fold model differences.
**Table S6:** Feature‐selection stability.
**Table S7:** Descriptive probability tertiles from random‐forest out‐of‐fold predictions.
**Table S8:** Predictor timing and availability audit.

**File S2:** Analysis code.

## Data Availability

Patient‐level clinical data are not publicly available because they contain information derived from hospital records. De‐identified analysis data may be made available from the corresponding author on reasonable request, subject to institutional approval, ethics requirements and any necessary data use agreement. The analysis scripts, fixed dependency versions and run instructions are provided as File S2; patient‐level data and derived record‐level predictions are not included in the code archive.
