# Supplementary material for: Explainable Machine Learning Models for Classifying Preoperative Anxiety in Patients With Femoral Neck Fractures: A Retrospective Cohort Study
Source: Nurs Open. 2026 Oct 16;13(10):e70919. doi: 10.1002/nop2.70919 (PMC13675517; doi:10.1002/nop2.70919)
Supplement: Supplementary file 1 [file NOP2-13-e70919-s001.docx]

**Supplementary File 1 Statistical Appendix**

This appendix reports the reproducible analysis after the predictor timing and administrative-field audit. The analysis dataset was read from a protected source file and was not modified.

## **Data and outcome definition**

The analytic file contained 271 surgical admissions involving 270 patients, including 79 admissions classified with preoperative anxiety. Available study documentation specified the binary outcome as HADS-A > 8. Raw HADS-A totals and item responses were not present in the analytic file; therefore, the score and threshold could not be verified against source records, reclassified using alternative cutoffs, or evaluated in a score-based sensitivity analysis.

## **Predictor availability and timing**

The intended model time point was the preoperative bedside assessment phase. The data source did not retain a field-level acquisition time for every candidate predictor, and HADS-A and several questionnaire measures were collected during the same assessment phase. The analysis is therefore an exploratory concurrent classification analysis, not a forecast of a later independent outcome. Case Order and OR Entry Time were excluded because they were administrative or operational fields with unclear availability at the intended assessment time. Serial number, identification number, patient identifier, name, surgery date and the outcome were also excluded. Thirty-two numeric candidate predictors remained.

## **Preprocessing, model development and validation**

No retained outcome or predictor values were missing or infinite. Fold-fitted median imputation and standardisation were retained in the pipeline, but no values were imputed. In every outer fold, LASSO selection, SMOTE, model tuning and Platt calibration were fit without access to the outer test fold. Five outer patient-grouped stratified folds were used, with a fixed random seed of 2022. Each outer training set was divided into fitting and calibration subsets; four inner patient-grouped folds and 18 random hyperparameter combinations were used for model selection. Random forest search was bounded to maximum depths of 3, 4 or 6 and minimum leaf sizes of 2, 4 or 8. This constraint was used to limit model complexity relative to the available 79 events.

## **Uncertainty and model comparisons**

All performance estimates were calculated from out-of-fold predictions. Percentile 95% confidence intervals used 2,000 patient-cluster bootstrap resamples. Pairwise model comparisons used paired patient-cluster bootstrap differences. Pointwise confidence intervals in Figure 3 are not simultaneous bands. The probability tertiles are descriptive and are not clinical thresholds.

Table S1. Data audit and analysis set

| **Item** | **Value** |
| --- | --- |
| Records | 271 surgical admissions |
| Unique patient groups | 270 |
| Preoperative anxiety events | 79 (29.2%) |
| Recorded variables | 40 |
| Retained candidate predictors | 32 numeric variables |
| Excluded operational fields | Case Order; OR Entry Time |
| Excluded identifiers | Serial number; ID number; Patient ID; Name; Surgery Date |
| Outcome | Preoperative Anxiety (binary 0/1; prespecified HADS-A > 8) |
| Missing or infinite retained values | 0 |
| Analysis target | Concurrent classification during the preoperative assessment phase |

Table S2. Prespecified model search ranges

| **Model** | **Search components** |
| --- | --- |
| XGBoost | 100-350 trees; learning rate 0.02-0.10; depth, child weight, sampling and regularisation tuned |
| Random forest | 300-800 trees; maximum depth 3, 4 or 6; minimum leaf size 2, 4 or 8; sampling and maximum features tuned |
| LightGBM | 100-350 trees; learning rate 0.02-0.10; leaves, depth, minimum child samples, sampling and regularisation tuned |
| Feature selection | LASSO inverse regularisation parameter tuned within inner grouped folds |

Table S3. Outer-fold data partitions

| **Outer fold** | **Fitting subset** | **Calibration subset** | **Test subset** |
| --- | --- | --- | --- |
| 1 | 163 (48 events) | 54 (15 events) | 54 (16 events) |
| 2 | 162 (47 events) | 54 (16 events) | 55 (16 events) |
| 3 | 162 (47 events) | 55 (16 events) | 54 (16 events) |
| 4 | 163 (48 events) | 54 (16 events) | 54 (15 events) |
| 5 | 162 (47 events) | 55 (16 events) | 54 (16 events) |

Table S4. Out-of-fold performance with patient-cluster bootstrap confidence intervals

| **Model** | **AUC (95% CI)** | **Brier score (95% CI)** | **Calibration intercept (95% CI)** | **Calibration slope (95% CI)** |
| --- | --- | --- | --- | --- |
| XGBoost | 0.921 (0.885-0.953) | 0.099 (0.074-0.124) | 0.028 (-0.397 to 0.447) | 0.907 (0.746-1.168) |
| Random forest | 0.932 (0.900-0.959) | 0.096 (0.075-0.118) | 0.041 (-0.325 to 0.386) | 1.017 (0.690-1.571) |
| LightGBM | 0.928 (0.894-0.957) | 0.096 (0.074-0.119) | -0.053 (-0.452 to 0.309) | 1.056 (0.847-1.355) |

Table S5. Paired out-of-fold model differences

| **Comparison** | **Metric** | **Mean difference** | **95% CI** |
| --- | --- | --- | --- |
| Random forest minus XGBoost | AUC | 0.011 | -0.011 to 0.033 |
| Random forest minus XGBoost | BRIER | -0.003 | -0.018 to 0.012 |
| LightGBM minus XGBoost | AUC | 0.007 | -0.013 to 0.029 |
| LightGBM minus XGBoost | BRIER | -0.003 | -0.017 to 0.012 |
| LightGBM minus Random forest | AUC | -0.004 | -0.027 to 0.019 |
| LightGBM minus Random forest | BRIER | -0.000 | -0.014 to 0.015 |

Table S6. Feature-selection stability

| **Feature** | **XGBoost** | **Random forest** | **LightGBM** |
| --- | --- | --- | --- |
| CD-RISC | 5/5 | 5/5 | 5/5 |
| MSPSS | 5/5 | 5/5 | 5/5 |
| SFQ | 5/5 | 5/5 | 5/5 |
| Frailty Index | 3/5 | 3/5 | 3/5 |
| Pre-op VAS Score | 3/5 | 2/5 | 3/5 |
| Familiar Faces | 3/5 | 4/5 | 4/5 |
| Education | 2/5 | 2/5 | 3/5 |
| Albumin (Alb) | 2/5 | 2/5 | 2/5 |

Table S7. Descriptive probability tertiles from random-forest out-of-fold predictions

| **Tertile** | **n** | **Observed events** | **Observed rate 95% CI** | **Predicted-probability range** |
| --- | --- | --- | --- | --- |
| Low | 91 | 0 (0.0%) | 0.0-4.1% | 0.0000-0.0435 |
| Middle | 90 | 13 (14.4%) | 8.6-23.2% | 0.0438-0.3687 |
| High | 90 | 66 (73.3%) | 63.4-81.4% | 0.3688-1.0000 |

Table S8. Predictor timing and availability audit

| **Field category** | **Handling in revised analysis** | **Reason** |
| --- | --- | --- |
| HADS-A outcome | Binary classification retained | Raw totals and items were unavailable; threshold sensitivity analysis was not possible. |
| Psychosocial questionnaires | Retained as concurrent-phase measures | Collected in the same preoperative assessment phase as HADS-A; not treated as prior predictors of a later independent outcome. |
| Clinical/EHR variables | Retained as candidate variables | Exact field-level acquisition timestamps were not preserved for every variable. |
| Case Order | Excluded | Administrative field with unclear availability at the intended assessment time. |
| OR Entry Time | Excluded | Operational field with unclear availability at the intended assessment time. |
| Direct identifiers and Surgery Date | Excluded | Administrative identifiers or record-level fields not used for modelling. |

Abbreviations: AUC, area under the receiver operating characteristic curve; CI, confidence interval; HADS-A, Hospital Anxiety and Depression Scale anxiety subscale; LASSO, least absolute shrinkage and selection operator; SMOTE, synthetic minority oversampling technique; VAS, visual analogue scale.
